# Dupuytren’s Contracture: Incidence of Injury-Induced Cases and Specific Clinical Expression

**DOI:** 10.3390/medicina56070323

**Published:** 2020-06-30

**Authors:** Gediminas Samulėnas, Rytis Rimdeika, Kęstutis Braziulis, Mantas Fomkinas, Rokas Paškevičius

**Affiliations:** 1 Department of Plastic and Reconstructive Surgery, Lithuanian University of Health Sciences, Eivenių str.2, LT 50009 Kaunas, Lithuania; kestutisbr@gmail.com; 2 Faculty of Medicine, Lithuanian University of Health Sciences, Eivenių str. 2, LT 50009 Kaunas, Lithuania; mfomkinas@gmail.com (M.F.); rokaspaskevicius@gmail.com (R.P.)

**Keywords:** Dupuytren’s contracture, acute injury, diathesis, false Dupuytren’s contracture-traumatic form, non-Dupuytren’s palmar fascial disease

## Abstract

*Background and objectives:* Dupuytren’s contracture is a chronic fibroproliferative hand disorder with a varying pattern of genetic predisposition across different regions and populations. Traumatic events have been found to have influence on the development of this illness and are likely to trigger different clinical forms of this disease. The aim of this study was to evaluate the phenomenon of development of Dupuytren’s contracture (DC) following an acute injury to the hand, and to observe the incidence and clinical diversity of such cases in daily clinical practice. *Materials and Methods:* We collected data of patients presenting with primary Dupuytren’s contracture in the Lithuanian population and evaluated the occurrence and clinical manifestation of this specific type of DC, arising following acute hand trauma. The diagnosis of DC was based on clinical signs and physical examination. Digit contractures were measured by goniometry, and the staging was done according to Tubiana classification. Injury-induced (injury-related) cases were identified using the “Criteria for recognition of Dupuytren’s contracture after acute injury” (established by Elliot and Ragoowansi). *Results:* 29 (22%) of a total of 132 cases were injury-induced DCs. Twenty-six of 29 patients in this group presented with stage I–II contractures. Duration of symptoms was 6 (SD 2.2) and 3.8 (SD 2.2) years in the injury-related and injury-unrelated DC groups, respectively. Mean age on the onset of symptoms in the injury-induced and non-injury-induced groups was 52 (SD 10.7) and 56 (SD 10.9), respectively. Patients from both groups expressed strong predisposition towards development of DC. *Conclusions:* Around one-fifth of patients seeking treatment for primary Dupuytren’s contracture seemed to suffer from injury-induced Dupuytren’s contracture. We noted that injury to the wrist and hand seems to trigger the development of less progressive Dupuytren’s contracture in younger age. Prospective randomized studies are required to confirm our findings.

## 1. Introduction

Dupuytren’s contracture (DC) is a chronic progressive fibroproliferative hand disorder of polygenetic and multifactorial origin, usually affecting middle-aged males. The standard course of this disease manifests as hard nodules in the palmar aponeurosis, leading to the formation of longitudinal fibrous bands. This results in contractures of the metacarpophalangeal (MCP) and/or proximal interphalangeal (PIP) joints, resulting in an extension deficit of one or more fingers, impairing hand function [1,2,3,4,5]. A specific form of Dupuytren’s contracture developing as a result of hand trauma has been observed and discussed previously [6,7,8,9,10,11,12,13,14,15]. The first publications describing this phenomenon introduced repetitive trauma as a possible cause [7]. Anderson was the first to expose an acute injury rather than continuous hand trauma to be the origin of this condition, and raised the idea of a different entity of Dupuytren’s contracture as a mild form of this illness [6]. However, some publications have reported conflicting data, suggesting that not all kinds of hand trauma are linked to the development of DC [16,17,18]. Later on, Rayan and Moore and Abe et al. came up with evidence supporting Anderson’s insights [19,20]. Furthermore, Elliot and Ragoowansi established a system of recognition of this distinct condition for further studies [7]. Definite conclusions on this topic still remain limited. We aimed to analyze the incidence of these cases in our practice and to evaluate the clinical expressions of this condition among Lithuanians. This is the first publication on this topic from our population, which exhibits a strong diathesis (tendency to develop DC) towards this pathology.

## 2. Materials and Methods

This study enrolled patients who were admitted to the Department of Plastic and Reconstructive Surgery, Hospital of LUHS, Kaunas Clinics for treatment of primary Dupuytren’s contracture. Approvals for this research were issued by the Department of Bioethics, Faculty of Public Health, LUHS (2017-10-23, BEC-MF-63) and Kaunas Regional Biomedical Research Ethics Committee (2019-03-08 No. BE-2-21). The diagnosis of DC was based on clinical signs and physical examination. Patient inclusion criteria: primary Dupuytren’s contracture of the specified hand. Demographic and behavioral patterns (smoking history, alcohol consumption habits), comorbidities, evidence of DC diathesis (family history, bilateral involvement, male gender, early onset (<50 years), ethnicity), detailed history of previous hand injuries and conditions, and duration of symptoms were acquired from patients and their medical documentation (records from general practitioners, extracts from the injury case histories with an objective evaluation of the hand, and records from the hospital’s database). Digit contractures (PIP and MP joints) were measured by goniometry, and the staging of DC was done according to Tubiana classification. Patients with multiple digit contractures were counted as a single case, taking the finger with the most expressed contracture as a reference. Patients with a positive history of trauma were evaluated using the “Criteria for recognition of Dupuytren’s. contracture after acute injury” (established by Elliot and Regoowansi, Table 1), creating injury-induced and non-injury-induced patient groups.

Data were analyzed using Statistical Package for Social Sciences (SPSS). Tests of normality (Shapiro–Wilk) displayed normally distributed continuous data, expressed in years. The chi-square test was used for categorical data. Independent-samples *t*-tests were used for continuous data comparisons. For hypothesis testing, the significance level of *p* < 0.05 was chosen.

## 3. Results

Between June 2017 and March 2019, a total of 132 patients were enrolled in the study: 109 males and 23 females, mean age 60 (SD 10.5), range 33–79. Mean age at the onset of DC was 55 (SD 11.0), range 25–76, duration of illness before surgery was 4.3 years (SD 2.4), range 0.5–10. Occupation of 70 (52%) patients was based on manual labor, and 56 (42%) patients had contracture in their dominant hand. In only 10 (8%) cases did the contracture not involve digits IV or V. Patient demographics and characteristics are summarized in Table 2.

### 3.1. Acute Trauma as a Trigger of Dupuytren’s Contracture

There were 73 patients with a history of acute trauma (fracture, laceration, surgery, ligament tears/sprains) in the hand or the distal third of the forearm of the affected hand. Twenty-nine (22%) cases matched the criteria and could be considered trauma-related or injury-induced cases of Dupuytren’s contracture. The remaining 44 patients were rejected mainly due to the timeframe from acute injury to the development of first symptoms exceeding 1 year.

The majority of injuries in the injury-induced DC group were fractures (14 cases), followed by 8 laceration injuries, 4 ligament tears, and 3 surgery-related cases (Table 3). Mean time from traumatic event until surgical treatment of DC and mean duration of DC symptoms in the injury-induced DC group were 6.7 (SD 2.3) and 6 (SD 2.2) years, respectively. The mean time from injury to first symptoms of DC was 0.7 (SD 0.3) years. Fourteen (48%) of 29 patients developed their first symptoms within 6 months.

We found statistically significant differences in age concerning the onset of illness. The mean age of onset of DC was 52 (SD 10.7) and 56 (SD 10.9) years in the injury-induced and non-injury-induced patient groups, respectively (*p* < 0.05). Twenty-four of 29 (83%) patients in the trauma-related DC group had single-digit involvement, whereas in the non-injury-related group, this occurrence was 67% (*p* > 0.05). Significantly lower counts of advanced contractures (Stages III, IV) were observed in the injury-induced DC group—3 of 29 (10%) in the injury-induced DC group and 41 of 103 (40%) in the non-injury-induced group (*p* < 0.05). There was also a difference in the duration of symptoms—6 (SD 2.2) and 3.8 (SD 2.2) years in the injury-induced and non-injury-induced DC groups, respectively (*p* < 0.05). Patients in the injury-induced DC group were more likely to have been younger when they developed symptoms of DC (*p* < 0.05). Longer duration of illness in relation to fewer expressed contractures in the injury-related group confirms a less progressive and milder course of the disease.

### 3.2. DC Diathesis

A high predisposition to DC was found, with all patients naturally possessing at least one factor of DC diathesis due to ethnicity. All patients were of Lithuanian descent, 109 (82.6%) were males, hereditary DC was observed in 39 (29.5%) cases, 71 (53.79%) patients exhibited bilateral involvement, and 40 (30.3%) patients had an early disease onset (Table 4). Findings were statistically different in reference to the early onset of DC symptoms.

Numbers of diathesis factors possessed per patient were compared between the injury-induced DC and non-injury-induced DC patient groups, providing no statistical significant difference. Groups did not differ in terms of diathesis (*p* > 0.05) (Table 5).

### 3.3. Interference in Daily Activities and Work Environment

DC was shown to create a burden in daily life for 90 (68%) patients, although pain did not seem to be the cause of the interference, affecting approximately one-third (34.8%) of patients (*p* > 0.05). There was a tendency towards a higher incidence of daily interference found among individuals whom worked in the manual labor sector (*p* = 0.07). However these findings were not statistically significant (Table 6).

Fifty-two patients (39.01%) patients were smokers >10 cigarettes/day, with a >5 year smoking history. High alcohol intake was discovered—the majority of patients (92, 69.7%) were consuming spirit beverages >2 times per month. There were nine (6.8%) patients with a medical history of diabetes mellitus; four patients with hepatic illness, one fatty-liver and three hepatitis B cases, respectively; and two patients with a diagnosis of epilepsy.

## 4. Discussion

Numerous authors have raised the idea of an association between traumatic events and the appearance of Dupuytren’s contracture since the 17th century, including Plater (1614), Goyrand (1835), B.G. Dupuytren himself (1833), and many others. Early evidence of this phenomenon first came in the form of case reports and small-sample studies [7]. This discussion even raised a different entity of DC-like conditions, which is still debated now. Anderson (1891) established a concept of “false Dupuytren’s contracture—traumatic form”. These clinical cases can be described as a mild course of DC, having single fibromatous bands, leading to less expressed contractures, being mostly unilateral, rarely requiring surgery, and having low chances of recurrence. Later on, Rayan and Moore (2005) raised a similar concept of “non-Dupuytren’s palmar fascial disease”, describing these cases as non-progressive unilateral palmar fascial proliferation without digital involvement among individuals with no family history and as being sex-unrelated [19]. In 2005, authors Elliot and Ragoowansi published a thorough literature review of 385 cases, including 52 cases from their own practice, establishing a system for recognition of Dupuytren’s contracture after acute injury [7]. Given the fact that a system for identification of injury-related DC was set, we took another direction and analyzed the incidence of cases matching the criteria of injury-induced DC among patients admitted for surgical treatment for Dupuytren’s contracture in a population with strong diathesis [21]. However, we did not evaluate the existence of Garrod pads. We cannot debate about rates of indication for surgery among patients with injury-induced DC, since all patients in our study were already admitted for treatment. The incidence of injury-induced Dupuytren’s contractures encountered in our practice corresponded to findings by Mikkelsen. Both populations express strong diathesis towards Dupuytren’s contracture [5]. Types of sustained injuries were similar to those reported by other authors [22,23,24].

The cases described in this study, albeit expressing a different course than standard Dupuytren’s contracture, did not fully align with the definitions offered by Anderson or Rayan and Moore [6,19]. We believe that the link between acute injury and the manifestation of Dupuytren’s contracture cannot be denied, although we found a disparity of clinical expression of DC among our cases and concepts described below. Our data suggest that injury-induced cases of DC lead to a less aggressive course than standard Dupuytren’s contracture, but to a lesser extent than is defined by the definitions of “false Dupuytren’s contracture-traumatic form” or “non-Dupuytren’s palmar fascial disease”. Anderson and Rayan proposed palm-limited fibromatous alterations mainly without finger involvement and contractures [6,19]. However, this was not the case among our patients. Other authors have also found a significant number of cases of injury-induced DC to develop as a standard form of the disease [7]. The possible cause of this discrepancy might be the diverse strengths of DC diathesis between different regions and populations [16]. Further studies are needed to confirm this finding.

As there was a difference in age on the onset of symptoms between the groups, there is a reason to believe that injury should be considered a provoking factor. Following this idea, the phenomenon of trauma can be defined purely as a trigger [22,23,24,25]. There is no reason to reject a likelihood of manifestation of a standard Dupuytren’s contracture in individuals with such strong inherited susceptibility in later years, without the impact of traumatic stimulus. This proposition has been supported by many authors [7].

It is usual in research into Dupuytren’s contracture that definite statements are sparse and many findings frequently raise more questions than answers, leaving a lot to research further. The background of the differences in disease severity still remains unclear. This study continued the discussion of the relationship between acute injury and the development of Dupuytren’s contracture.

Study limitations: The results of our study support this association, but must be interpreted with caution due to the biases related to the retrospective design of our investigation. Our study demonstrated high clinical variability among injury-induced cases of DC. A second limitation of our study was the omission of Garrod pads from the list of predisposing factors. Third, the single-center setting and limited number of patients preclude generalizability of our study results. Further prospective randomized studies evaluating different causes of Dupuytren’s contracture are required to confirm our findings.

## 5. Conclusions

Around one-fifth of patients seeking treatment for primary Dupuytren’s contracture seemed to suffer from injury-induced Dupuytren’s contracture. We noted that injury to the wrist and hand seems to trigger development of less progressive Dupuytren’s contracture at a younger age. Prospective randomized studies are required to confirm our findings.

## Figures and Tables

**Table 1:** Criteria for recognition of Dupuytren’s contracture after acute injury.

Criteria for Recognition of Dupuytren’s Contracture after Acute Injury (Elliot and Ragoowansi, 2004)
There is objective evidence of injury with no evidence of Dupuytren’s disease before the injury; The injury was within the same hand, wrist, or forearm as the first hand to develop disease; The patient may be of any age and may or may not exhibit conditions predisposing to Dupuytren’s disease or indicative of a diathesis; Disease appears within 1 year of injury; A single nodule or band appears first in the palm of the injured hand; Disease commonly remains limited to the part of the hand initially involved but may progress within the same hand or to the other hand, and may occasionally become significant in degree.

**Table 2:** Demographics and patient characteristics.

	All Patients	Injury-Induced DC *	Non Injury-Induced DC	*p*
Total No. of patients	132	29	103	-
Sex (M/F)	109/23	24/5	85/18	>0.05
Age (on the day of surgery)	60 (SD 10.5)	58 (SD 10.6)	60 (SD 10.5)	>0.05
Age at the onset of DC	55 (SD 11.0)	52 (SD 10.7)	56 (SD 10.9)	<0.05
Duration of DC symptoms	4.3 (SD 2.4)	6 (SD 2.2)	3.8 (SD 2.2)	<0.05
Manual labor	70	14	56	>0.05
Stages				<0.05
0–I–II	88	26	62	
III–IV	44	3	41	
No. of digits affected (per patient):				>0.05
Only nodules	7	1	6	
1	93	24	69	
2	24	3	21	
3	8	1	7	
Digits affected				-
I	2	1	1	
II	2	0	2	
III	15	2	13	
IV	72	15	57	
V	68	16	52	

* patients, matching the Criteria for Recognition of Dupuytren’s Contracture after Acute Injury (Table 1).

**Table 3:** Characteristics of traumatic injuries.

**Laceration injuries**	**8**	1 forearm, 5 palmar, 2 digit lacerations
**Fractures (total)**	**14**	
Wrist bones	9	8 distal radius fractures, 1 scaphoid fracture
Metacarpal bones	5	5 MCB crush fractures
**Surgery**	**3**	2 CTR, 1 TRF
**Ligament tears/sprains (total)**	**4**	
Wrist	3	3 hyperextension injuries
Thumb	1	MCP joint luxation injury
**Total**	29	

MCB—metacarpal bone, CTR—carpal tunnel release, TFR—trigger finger release, MCP—metacarpophalangeal.

**Table 4:** List of diathesis factors.

Diathesis Factors	No. of Patients	Injury-Induced DC *	Non-Injury-Induced DC	*p*
First-degree relative with DC	39 (29.6%)	8	31	>0.05
Age of onset < 50	40 (30.3%)	13	27	<0.05
Bilateral involvement	71 (53.8%)	16	55	>0.05
Male sex	109 (82.6%)	24	85	>0.05
Caucasian ethnicity (all)	132 (100%)	29	103	-

* patients, matching the Criteria for Recognition of Dupuytren’s Contracture after Acute Injury (Table 1).

**Table 5:** Diathesis factors per patient.

No. of Diathesis Factors Per Patient	No. of Patients	Injury-Induced DC	Non-Injury-Induced DC
1	9 (23.5%)	1	8
2	31 (30.3%)	4	27
3	57 (28.8%)	16	41
4	26 (15.9%)	7	19
5	9 (1.5%)	1	8

**Table 6:** Impact of DC in daily life.

	Manual Labor	Pain	Interference in Daily Activities
Positive	70 (53.0%)	46 (34.8%)	90 (68.2%)
Negative	62 (47.0%)	86 (65.2%)	42 (31.8%)
Total (all patients)	132	132	132
